# Less pain reported 5 years after cementless compared to cemented unicompartmental knee replacement: an analysis of pain, neuropathy, and co-morbidity scores

**DOI:** 10.1007/s00167-023-07589-4

**Published:** 2023-09-30

**Authors:** Azmi Rahman, Benjamin Martin, Cathy Jenkins, Hasan Mohammad, Karen Barker, Christopher Dodd, William Jackson, Andrew Price, Stephen Mellon, David W Murray

**Affiliations:** 1 Nuffield Department of Orthopaedics, Rheumatology, and Musculoskeletal Sciences, University of Oxford, Oxford, UK; 2 Nuffield Orthopaedic Centre, Oxford University Hospitals NHS Foundation Trust, Oxford, UK

**Keywords:** Pain, Knee replacement, Unicompartmental, Arthroplasty, Replacement, Knee, Cemented, Cementless, Patient-reported outcome measures

## Abstract

**Purpose:**

To compare patient-reported pain scores and assess the influence of neuropathy and co-morbidity, on knee pain following cemented and cementless medial unicompartmental knee replacement (UKR) 5 years after surgery.

**Method:**

In this longitudinal study, 262 cemented and 262 cementless Oxford UKR performed for the same indications and with the same techniques were recruited. Patients were reviewed at five years, evaluating patient-reported pain and association with clinical outcomes. Intermittent and Constant Osteoarthritis Pain (ICOAP), PainDETECT (PD), Charnley score, Oxford Knee Score (OKS) and American Knee Society Score (AKSS) were compared.

**Results:**

In both cohorts, intermittent pain was more common than constant pain (47% vs 21%). Cementless knees reported significantly less pain than cemented (ICOAP-Total 5/100 vs 11/100, *p* < 0.0001). A greater proportion of cementless knees experienced no pain at all (ICOAP = 0/100, 61% vs 43%, *p* < 0.0001) and 75% fewer experienced severe or extreme pain. Pain sub-scores in PD, OKS and AKSS follow this trend. Pain was unlikely to be neuropathic (PD positive: 5.26%), but patients reporting high levels of ‘strongest’ pain were three times more likely to be neuropathic. Patients with co-morbidities (Charnley C) experienced greater pain than those without (Charnley A+B) across all knee-specific scores, despite scores being knee specific.

**Conclusion:**

Both cemented and cementless UKR in this study had substantially less pain than that reported in literature following TKR. Cementless UKR had significantly less pain than cemented UKR in all scores. Two-thirds of patients with a cementless UKR had no pain at all at 5 years, and pain experienced was most likely to be mild and intermittent with no patients in severe or extreme pain. Patients with cementless UKR that had higher levels of pain were more likely to have co-morbidity or evidence or neuropathic pain. It is unclear why cementless UKR have less pain than cemented; further study is necessary.

## Introduction

Unicompartmental knee replacement (UKR) is an alternative to total knee replacement (TKR) for the surgical management of antero-medial osteoarthritis. The UK and other registry data show that UKR offers better function, faster recovery with fewer and less severe complications and is more cost effective [2, 3, 12, 16]. However, UKR has a higher revision rate than TKR, in part due to aseptic loosening [20]. To reduce the incidence of aseptic loosening, the cementless Oxford UKR (Zimmer Biomet), with a porous bone–implant interface for bony ingrowth to improve fixation, was developed. UK registry data have shown that the aseptic loosening rate of the cementless Oxford UKR is half that of the cemented [2].

Small randomised controlled trials designed to assess radiographic outcomes have suggested that patient-reported outcomes (PROMS) might be better following cementless than cemented Oxford UKR; however, these studies were underpowered for assessing clinical outcomes [13, 24]. In an adequately powered 5-year study, it was found that cementless Oxford UKR had better quality-of-life (EQ-5D), Oxford Knee Scores (OKS) and American Knee Society Scores (AKSS) than cemented [17]. Analysis of the sub-scores of these measures suggested that the improvement was due to a reduction in pain with the cementless UKR. However, it is not clear how large the difference is or why it might be present.

Following knee replacement, pain has traditionally been assessed with a few questions that form part of the overall assessment. Examples are the pain scores with the OKS or the AKSS [9, 11]. There are other scores available that are more sensitive and assess pain in much more detail. ICOAP (Intermittent and Constant Osteoarthritis Pain) is a PROM designed to assess pain in knee osteoarthritis [22]. PainDETECT (PD) is a PROM designed to assess likelihood of pain being neuropathic in origin, and also includes three visual analogue scales [6]. It is also possible that knee pain may not actually be arising from the knee and is a manifestation of pathology elsewhere.

This aim of this study is to compare the magnitude and nature of pain five years following cemented and cementless Oxford UKR, with the hypothesis being that there is no difference in pain. This will be done using specific pain scores, and the influence of co-morbidity and neuropathic pain will also be explored.

## Methods

A longitudinal cohort study comparing 5-year outcomes of cemented and cementless medial mobile-bearing Oxford UKR implanted from 2006 to 2012 was conducted. All procedures were performed by four high-volume knee surgeons at two hospitals in the United Kingdom. During the period of this study, clinicians transitioned from use of the cemented Oxford UKR to the cementless Oxford UKR. The indications, pre-operative care and post-operative care were identical for both procedures. Surgical technique and instrumentation were identical [1] except for method of fixation—wider slots were produced to accept a cement mantle interface in cemented and narrower slots and holes were produced for interference fit in cementless. ‘Hybrid’ implants were not included. Patients were reviewed by independent research orthopaedic physiotherapists pre-operatively and 5 years post-operatively. Patients who were unable to be reviewed in clinic were contacted by post or telephone to obtain patient-reported outcomes. Revision rates were also tracked.

Patients were assessed five years post-operatively using two pain-specific instruments: the *Intermittent and Constant Osteoarthritis Pain (ICOAP*) instrument and *painDETECT* instrument. Pain sub-scores for the Oxford Knee Score (*OKS*) and the *American Knee Society Score (AKSS*), as well as Charnley classification, were analysed.

The knee-specific *ICOAP* score quantifies the magnitude and nature of knee pain [22]. *ICOAP-A* assesses constant pain and *ICOAP-B* assesses intermittent pain, i.e. pain that *“comes and goes”*. *ICOAP-A* is the total score from five questions, and *ICOAP-B* from six questions. Each question is equally weighted, and scores pain in different contexts from 0 (‘no pain’) to 4 (‘extreme’). *ICOAP-Total* is the sum of both A and B scores. *ICOAP-A, -B*, and -*Total* are scaled and scored between 0 and 100, with a higher score indicating worse pain. Patients scoring 0 on all scores were considered to have “no pain at all”.

The *painDETECT* instrument includes three visual analogue scales (*PD-VAS*) assessing ‘*Now’*, ‘*Average*’ and ‘*Strongest*’ pain experienced over the last four weeks and a separate ‘*PD-Q*’ score to assess likelihood of the pain being neuropathic. The *PD-VAS* are scored between 0 and 10 (increasing with magnitude of pain), and *PD-Q* is between 0 and 38, where *PD-Q* ≤ 12 is negative, 13 ≤ *PD-Q* ≤ 18 is unclear and *PD-Q* ≥ 19 is positive for a neuropathic pain component [6].

The *OKS-Pain* sub-score (0–20, higher is better) [9] and *AKSS-Pain* sub-score (non-continuous score of 0/10/20/30/40/45/50, higher is better) [11] were also analysed.

Patients were also classified into Charnley classification [5]: a 3-point classification of disease co-morbidity—A: single knee affected, B: both knees affected and C: multiple arthritis or medical infirmity.

Patients were recruited to the study and implanted with the cemented or cementless versions of the Phase 3 Oxford Partial Knee, in a non-blinded manner. Pre-operatively, patient demographics (date-of-birth, sex, height, weight), *OKS-Pain* and *AKSS-Pain* were compared between cohorts to ensure an even baseline of patients were recruited into both groups. Five-year post-operative *ICOAP*, *painDETECT*, *OKS-Pain* and *AKSS-Pain* measures were collected and analysed. Differences in magnitude and nature of pain between cemented and cementless cohorts were assessed, with the added impact of patients’ Charnley classification.

### Statistics

All datasets were assessed for normality (Shapiro–Wilk). Significances were assessed using unpaired Student *t*-tests (where parametric) and Mann–Whitney U-tests (where non-parametric). Discrete categories were assessed with Chi-square tests, or Chi-square test for trend, where appropriate. Statistical significance is defined by p-values of < 0.05. Where multiple scores are compared, score ranges are converted to 0–100, with 0 being worst and 100 being best. Data were analysed and visualised using GraphPad Prism (GraphPad Software, San Diego, California, USA) and Excel (Microsoft, Redmond, Washington, USA).

### Recruitment

Patients who underwent a UKR during the study period were recruited to the study and followed up at 5 years (mean 5.06, SD 0.29) if they did not have a revision to a TKR. At 5 years, 524 knees were asked to complete the questionnaires. Post-operative *ICOAP*, *painDETECT PD-VAS* and additional *painDETECT PD-Q* were completed for 487 (92.9%), 470 (89.6%) and 394 (75.2%) knees, respectively. Post-operative *OKS-Pain*, *AKSS-Pain* and Charnley classification scores were collected for 524 (100%), 419 (80.0%) and 436 (83.2%) of knees. Knees without data are due to lack of patient response or incomplete responses. Paired post-operative *ICOAP* and *OKS-Pain* scores were available for 487 (92.9%) of knees, and paired post-operative *ICOAP* and *AKSS-Pain* scores were available for 386 (73.7%) of knees. Ninety four patients in this study had UKR bilaterally, and their knees were studied independently where possible by outcome measure usage guidelines.

During this study, cemented UKR was performed earlier (median: 2008) compared to cementless UKR (median: 2011), albeit with substantial overlap. Sub-cohort analysis was performed to assess the impact of this non-contemporaneity, comparing early and late sub-groups to assess if notable differences arose.

## Results

### Baseline

No significant differences were noted between the cemented and cementless cohorts with respect to pre-operative age, gender, height, weight and body mass index (Table 1). There were no significant differences among the pre-operative *OKS-Pain*, *AKSS-Pain* or Charnley Grade.

**Table 1:** Pre-operative characteristics of cemented and cementless cohorts

	Cemented (*n* = 267)		Cementless (*n* = 278)		
	No. of values	Mean (SD)	No. of values	Mean (SD)	*p*-value
Age (years)	267	65.6 (9.8)	278	65.50 (11.7)	0.935^a^
Height (cm)		169 (10.3)		171 (9.7)	0.118^a^
Weight (kg)		85.4 (16.9)		85.8 (15.5)	0.787^a^
BMI		29.8 (5.2)		29.4 (4.3)	0.379^a^
Sex		57.7% male		62.7% male	0.209^b^
		42.3% female		37.1% female	
OKS (Pain)	176	9.31 (3.87)	191	9.23 (3.74)	0.724^a^
AKSS (Pain)	163	16.5 (14.7)	162	14.3 (13.9)	0.173^a^
Charnley Grade	37	4 A	198	37 A	0.314^b^
		14 B		83 B	
		19 C		78 C	

All distributions were determined to be non-parametric (Shapiro–Wilk *p* < 0.05)

^a^Mann–Whitney U Test

^b^Chi-Square Test

### Revision

Within 5 years after surgery, the cemented cohort had 1 conversion to TKR and 1 bearing replacement, and the cementless cohort had 2 conversions to TKR. Conversion to TKR in all 3 cases listed were due to lateral progression of disease.

### Magnitude and nature of pain

**Fig. 1:**
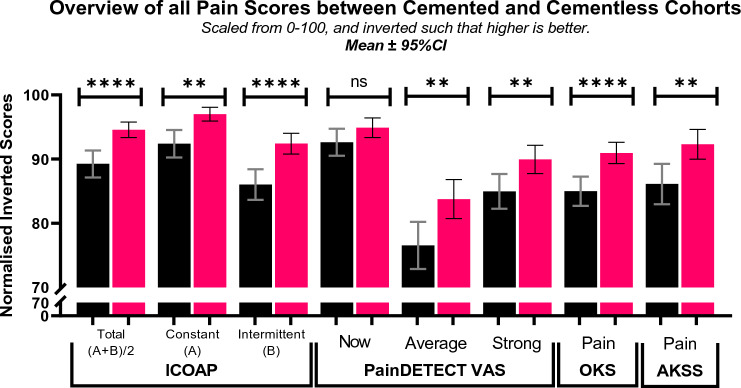
Mean ± 95% CI scores ICOAP, PainDETECT, OKS-Pain and AKSS-Pain scores, for cemented and cementless cohorts. Cemented cohort data is in black, and cementless cohort data is in red. All scores scaled from 0 to 100 and inverted where necessary such that higher is better (ICOAP-A and -B inverted, painDETECT multiplied by a factor of 10 from 0–10 to 0–100 and inverted, OKS-Pain multiplied by a factor of 5 from 0–20 to 0–100, AKSS-Pain multiplied by a factor of 2 from 0–50 to 0–100)

Both cemented and cementless cohorts reported low pain and the cementless cohort had lower pain than the cemented in all pain measures. Cementless reported lower in *ICOAP-Total* (5.4 vs 10.8, *p* < 0.0001, lower is better), stemming from lower *ICOAP-A-Constant* and *ICOAP-B-Intermittent* (2.98 vs 7.60, *p* = 0.0014 and 7.58 vs 14.0, *p* < 0.0001, respectively). For *PD-VAS*, cementless reported lower *PD-VAS-Average* and *PD-VAS-Strong* but similar *PD-VAS-Now* pain (1.00 vs 1.50 *p* = 0.0011, 1.62 vs 2.34 *p* = 0.0022, 0.51 vs 0.74 *p* = 0.1219). *OKS-Pain* and *AKSS-Pain* were lower in the cementless cohort (18.2 vs 17 *p* < 0.0001, 46.2 vs 43.1 *p* = 0.0046, higher is better) (Fig. 1).

**Fig. 2:**
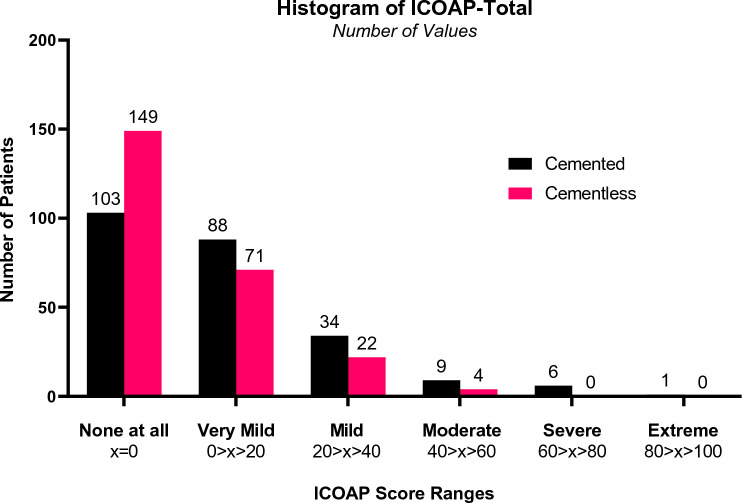
Histogram of 5-year ICOAP (total) score, for cemented and cementless cohorts. Two-sided Chi-Square Test across all categories had a significant difference of *p* = 0.0006. Two-sided Chi-Square Test between ‘x = 0’ and other categories had a significant difference of *p* < 0.0001

*ICOAP-Total* scores were categorised into increasing magnitudes of pain from “*None at all” (ICOAP-Total* = 0) to *Extreme*. The cementless cohort had a greater proportion of scores in the *None at all* category and the cemented had a greater proportion in all other categories (Chi-Square, *p* = 0.0006). 61% of the cementless cohort compared to 43% of the cemented cohort (*p* < 0.0001) reported *None at all*. 7 (2.9%) of the cemented cohort had *Severe* (60–80 out of 100) or *Extreme* (80–100 out of 100) pain, whereas none of the cementless did. (Fig. 2).

**Fig. 3:**
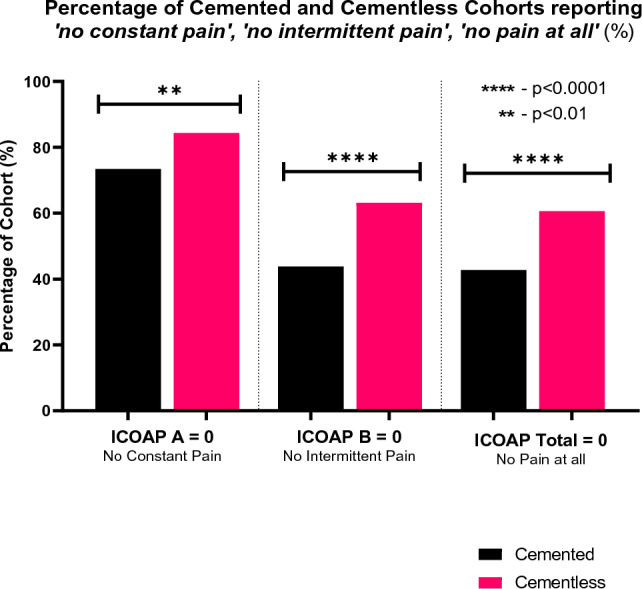
Percentage of 5-year ICOAP-A/B scores with score 0 of 100, in cemented, cementless and combined cohorts

Patients who scored *ICOAP-Total* = 0 report no constant or intermittent pain (*ICOAP-A* = *ICOAP-B* = 0). A significantly greater proportion of the cementless cohort reported having *‘no constant pain at all’* compared to the cemented cohort (84% vs 73%, *p* = 0.003), and *‘no intermittent pain at all’* (63% vs 44%, *p* < 0.0001); the difference was greater for intermittent than constant pain. In both cohorts, the proportion of patients having *‘no intermittent pain at all’* was near-identical to those having ’n*o pain at all’* (*ICOAP-B* = 0, 44% vs 63%, *ICOAP-Total* = 0, 43% vs 61%), suggesting that if any patient experienced constant pain, they would also experience intermittent pain (Fig. 3).

**Fig. 4:**
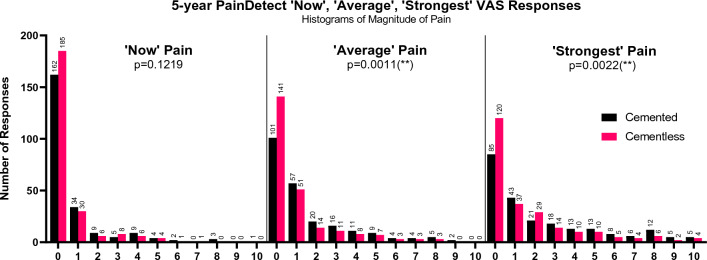
Histograms of 5-year ‘painDETECT Scales’ scores. *p*-values given for Mann–Whitley U-Tests performed between scores for cemented and cementless cohorts

In the *PD-VAS*, 0 (indicating ‘no pain’) was the most frequently selected score in all scales for both cohorts (Fig. 4).

### Neuropathic pain

The *painDETECT PD-Q* score screens for the presence of a neuropathic component to pain. *PD-Q* ≤ 12 is considered negative with patient unlikely (< 15%) to be neuropathic, while *PD-Q* ≥ 19 is considered positive with > 90% likelihood of neuropathy [6]. Patients were categorised into pain categories based on *PD-VAS-Strongest* scale response, from *No* to *Extreme* pain. Across both cohorts, where there is pain, it is highly likely to be nociceptive (*PD-Q* ≤ 12 in 88%). None of the patients experiencing no pain (*PD-VAS-Strongest* = 0/10) scored positive for neuropathic pain (cemented + cementless, 0% *PD-Q* ≥ 19). Patients experiencing *Severe/Extreme* pain (*PD-VAS-Strongest* ≥ 7/10) were 3.5–3.8 times more likely to be neuropathic (% of cohort with *PD-Q* ≥ 19 in *PD-VAS-Str* ‘7–10’ vs ‘1–6’) (Table 2).

**Table 2:** Likelihood of having a neuropathic component to pain (based on PainDETECT PD-Q score) at different pain levels, scored on the PainDETECT VAS Strongest scale. Results (n and %) from cemented and cementless cohorts

		Cemented			Cementless		
		PD-VAS-Str = 0	PD-VAS-Str = 1–6	PD-VAS-Str = 7–10	PD-VAS-Str = 0	PD-VAS-Str = 1–6	PD-VAS-Str = 7–10
		No Pain	Very Mild to Moderate	Severe to Extreme	No Pain	Very Mild to Moderate	Severe to Extreme
PD-Q negative (≤ 12)	*n*	49	14	0	75	10	0
	%	71.0	13.9	0.0	77.3	10.9	0.0
PD-Q unclear (13–18)	*N*	20	82	19	22	80	11
	%	29.0	81.2	82.6	22.7	87.0	91.7
PD-Q positive (≥ 19)	*n*	0	5	4	0	2	1
	%	0.0	5.0	17.4	0.0	2.2	8.3
Total	*n*	69	101	23	97	92	12
	%	100.0	100.0	100.0	100.0	100.0	100.0

### Pain and co-morbidity

**Fig. 5:**
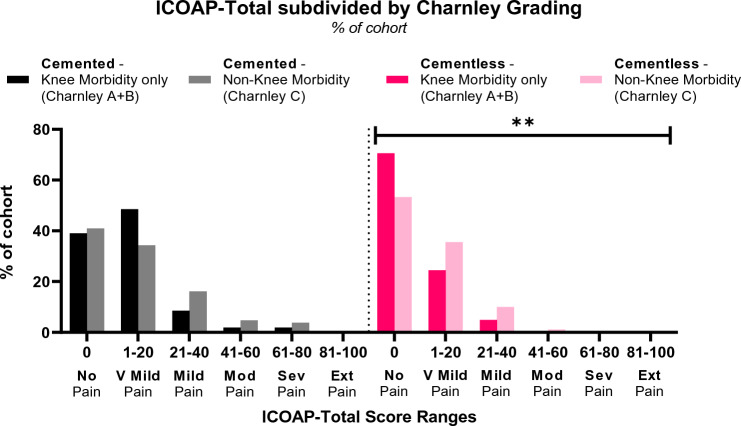
ICOAP-Total Scores in cemented and cementless cohorts, split by Charnley grades A+B (knee arthritis only) and Charnley C (non-knee morbidity). V Mild: Very Mild, Mod: Moderate, Sev: Severe, Ext: Extreme. **—*p* < 0.001

**Fig. 6:**
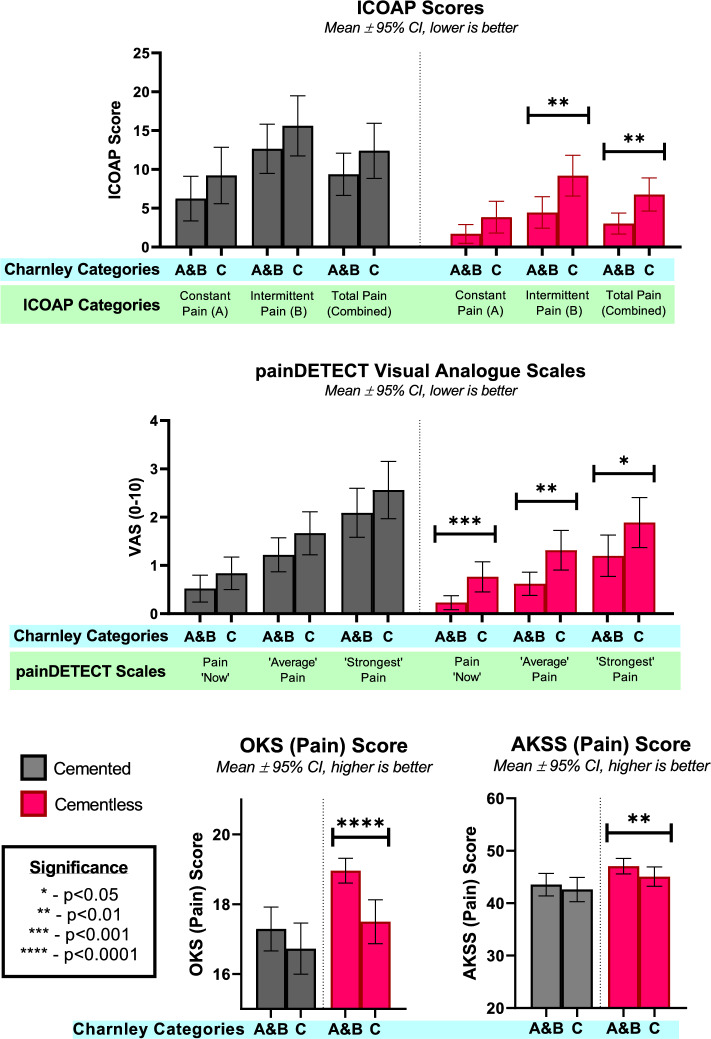
Mean (± 95% CI) responses to ICOAP, painDETECT VAS, OKS (Pain), and AKSS (Pain) categorised into Charnley scores. All distributions are non-parametric (Shapiro–Wilk *p* < 0.05), and differences tested with Mann–Whitley U test

In both cohorts, the patients with non-knee morbidities (*Charnley C*) report greater pain (*ICOAP-Total*) than patients with knee arthritis only (*Charnley A*+*B*) (Fig. 5), with approximately double the proportion of *Charnley C* patients experiencing *Mild* or greater pain than *Charnley A* + *B* patients (*ICOAP-Total* > 20 in *Charnley A*+*B* vs *Charnley C*, 12.4 vs 24.8 in cemented, 4.9 vs 11.1 in cementless). This trend was significant in the cementless cohort (Chi-Square *p* = 0.0092), but not in cemented (*p* = 0.1902) (Fig. 5). These differences are consistent with comparisons of mean scores between the cemented and cementless cohorts across *ICOAP*, *PainDETECT*, *OKS-Pain* and *AKSS-Pain* scores (Fig. 6).

### Non-contemporaneity between procedures

**Fig. 7:**
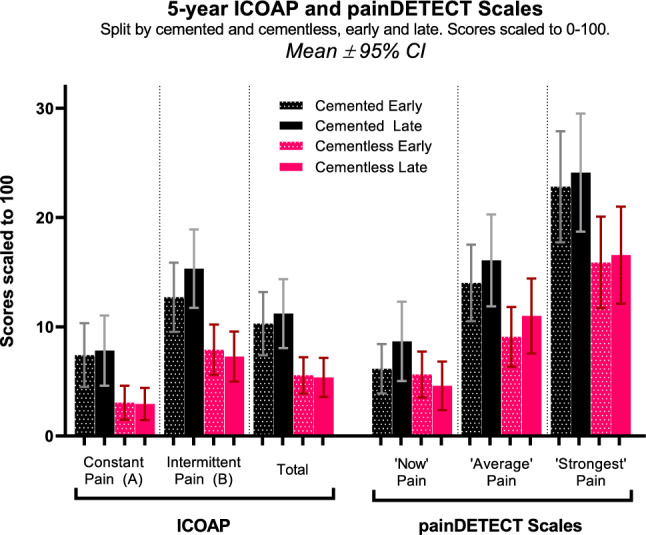
Mean ± 95% CI scores for ICOAP and painDETECT scales, for cemented and cementless cohorts, further classified into early and late sub-groups. All scores scaled from 0 to 100 (ICOAP already 0–100, painDETECT scales multiplied by a factor of 10 from 0–10 to 0–100)

The cemented procedures were performed, on average, 3 years earlier than the cementless procedures. To assess if the earlier differences noted were due to this non-contemporaneity, all tests were repeated, splitting cohorts into early and late sub-groups across their medians (cemented 01/2008, cementless 07/2011). There were no differences between early and late cohort sub-groups. Any differences found between *late-cemented* and *early-cementless* groups, which were approximately contemporaneous, are consistent with those found in the overall cohorts (Table 3**, **Fig. 7).

**Table 3:** Significance tests between early and late sub-groups for cemented and cementless cohorts, for all mean comparison tests performed in this study. P values < 0.05 (significant) have been highlighted in bold

*p*-values for groups compared	Test	Early cemented	Early cementless	Late cemented	Cemented
		Late cemented	Late cementless	Early cementless	Cementless
ICOAP-Total, mean scores	U	0.6464	0.9045	**0.0028**	** < 0.0001**
ICOAP-A, mean scores	U	0.9665	0.8581	**0.0217**	**0.0014**
ICOAP-B, mean scores	U	0.2710	0.8064	**0.0006**	** < 0.0001**
ICOAP-Total, pain categories	Chi-Square	0.6736	0.8965	**0.0011**	**0.0006**
ICOAP-Total, pain vs no pain (0 vs non-0)	Chi-Square	0.8671	0.9588	**0.0048**	** < 0.0001**
ICOAP-A, pain vs no pain (0 vs non-0)	Chi-Square	0.9461	0.8186	**0.0296**	**0.0030**
ICOAP-B, pain vs no pain (0 vs non-0)	Chi-Square	0.5178	0.9613	**0.0009**	**0.0001**
painDETECT ‘now’ scale, mean scores	U	0.7126	0.1257	0.8681	0.1219
painDETECT ‘average’ scale, mean scores	U	> 0.9999	0.8483	**0.0216**	**0.0011**
painDETECT ‘strongest’ scale, mean scores	U	0.9713	0.8477	**0.0392**	**0.0022**
painDETECT PD-Q score, positive vs negative	Chi-Square	0.2268	0.9937	0.0792	0.1554
OKS-Pain (mean scores)	U	0.3474	0.8922	**0.0012**	** < 0.0001**
AKSS-Pain (mean scores)	U	0.6850	0.9105	**0.0328**	**0.0046**

**U**: Mann–Whitney U Test. Distributions tested were non-parametric (Shapiro–Wilk *p* < 0.05)

**Chi-Square**: Two-tailed Chi-Square Test

## Discussion

This study provides compelling evidence that patients experience low levels of pain following both cemented and cementless UKR. A large proportion of both cohorts reported no pain, and in cases with more serious pain, much of it did not arise from the knee. The amount of pain following cemented or cementless UKR was markedly less than that reported in the literature following TKR [2, 3, 12, 16]. At five years, the cementless UKR was found in all the scores we used to have significantly less pain than the cemented UKR despite the floor and ceiling effects resulting from the very low levels of pain (Fig. 2).

As mean pain scores were low for both cohorts, the distributions of the scores offer important insights. When compared to cemented UKR, patients with the cementless UKR were 45% more likely to have *No pain at all* (43% vs 61%). No cementless cases had *Severe* or *Extreme* pain, whereas 2.9% of the cemented did. As a result, most cases had *No*, *Very Mild* or *Mild* pain (cementless 98%, cemented 93%). This is corroborated by the revision rate of 0.76% in both cohorts, where there were no revisions for unexplained pain.

The different scores also give insight into differences in the nature of pain. Pain was twice as likely to be intermittent than constant in both cohorts (*ICOAP-B* > 0 47% vs *ICOAP-A* > 0 21%), a trend corroborated in literature [4, 15, 19, 26]. In general, patients reporting constant pain, also reported intermittent pain. Both pain scores were significantly, but proportionally, lower for the cementless cohort. The *painDETECT VAS* results followed the expected trend—‘*Strongest’* pain was worse than the ‘*Average’* which was worse than pain ‘*Now’* (1.97 vs 1.25 vs 0.621, out of 10). The cementless cohort reported less pain across the 3 groups than the cemented. The difference was statistically significant for ‘*Strongest’* and ‘*Average’*, but not for ‘*Now’* Pain, which may be explained by the few patients reporting any pain ‘*Now’*, causing an especially strong floor effect (Fig. 4).

Across both cohorts, where there is pain, it is highly likely to be nociceptive (*painDETECT PD-Q* ≤ 12 in 87.8%), i.e. with a likely significant physiological origin. However, if the worst pain that patient experienced was *Severe/Extreme* (*PD-VAS-Strongest* ≥ 7/10) rather than *Very Mild/Mild/Moderate* (*PD-VAS-Strongest* ≥ 1–6), those patients were 3.5–3.8 times more likely to have a neuropathic element to their pain (Fig. 5). This is corroborated by the influence of *Charnley* grades on pain scores; despite all scores being knee-specific, patients with multiple arthritis or medical infirmity (*Charnley C*) consistently scored more poorly than those with only arthritis in the knees (*Charnley A*+*B*) (Fig. 6). More importantly, patients reporting higher levels of pain in *ICOAP* are markedly more likely to be *Charnley* *C* patients (Fig. 5). Interestingly, these differences were statistically significant in the cementless, but not cemented, cohort. This may be due to the greater “knee-origin” pain with the cemented cohort masking “other-origin” pain, while the cementless cohort experience weaker “knee-origin” pain, thereby allowing the relatively stronger influence of the “other-origin” pain to increase scores in *Charnley C* patients.

These correlations with neuropathy and non-knee morbidity suggest that pain perceived by patients to be knee-specific may not necessarily be caused by knee pathology, but instead be of external origin, be it biological, neuropathic, or psychosocial, as discussed in literature [10]. These external causes appear to be most likely in patients experiencing higher levels of pain, whilst “knee-origin” pain appears in most cases to be mild.

The origin of pain in UKR and the cause of the differences in pain between cemented and cementless implants remain unclear. The main difference between the implants is the bone–implant interface, which is likely to be responsible for the difference in pain. There are fewer radiolucent lines under cementless than cemented tibial components [14, 21, 23]. However, it has been shown that, following cemented fixation, there is no relationship between pain and radiolucency [7]. An alternative explanation relates to the stress within the tibial condyle, which increases appreciably following UKR and despite remodelling, this might remain elevated and contribute to pain [28]. The main reason for this is removal of the subchondral bone plate, which acts as a tension band supporting the medial condyle. If tension was transmitted between the wall and tibial eminence, this tension band may be at least partially restored and there should be less pain [28]. In this region, there are less radiolucencies with cementless than cemented components [25]. Further study is needed to understand why the pain occurs [18].

Scores from both cemented and cementless UKR cohorts are better than TKR scores in the literature. *OKS-Pain* in TKR at 5 years is reported to be 15.9 [27], which is poorer than cemented and cementless UKR scores in this study (17.0, 18.2 of 20). The differences are greater with the pain-specific score ICOAP and exceed minimally important clinical difference (MCID 18 of 100) [29]: UKR patients experience 8 times less constant pain (*ICOAP-A* 5.27 vs 42.3), 5 times less intermittent pain (*ICOAP-B* 10.8vs52.3), and 6 times less total pain (*ICOAP-Total* 8.06 vs 47.7) than TKR [4]. These studies are not matched and have different lengths of follow up. Nonetheless, TKR scores are consistent in literature [19, 29], do not change substantially after 6 months [10], and any changes that do occur would likely be small relative to the differences noted.

Score differences between cohorts in this study are not greater than published MCIDs: *ICOAP-Total* 18/100 [29], PD-VAS 0.9/10 [30]. However, these MCIDs were developed for TKRs, where scores are more normally distributed than the UKR scores encountered in this study. Due to strong floor/ceiling effects (Figs. 3, 5, 6), it is unfeasible to apply them to this study: for example, the cemented *ICOAP-Total* was 10.8, which cannot be decreased by 18 points (the MCID). MCID for *OKS-Pain* and *AKSS-Pain* were not found in literature.

The main limitations of the study are that it is not randomised and the cemented cohort was implanted predominantly before the cementless across the 6-year study period. Therefore, differences in pain observed could be related to improvements in surgical practice over time. However, when comparing the *early* and *late* sub-groups within the *cemented* and *cementless* cohorts, no significant differences are found. In contrast, when comparing the *late-cemented* and *early-cementless* sub-groups, which were implanted at approximately the same time, differences were fully consistent with the overall cemented and cementless groups. This demonstrates little, if any, effect of the date of implantation on outcome. In addition, the instrumentation and implantation procedure are largely identical. Hence, differences between the two cohorts are most likely due to differences in the implant itself, rather than other confounding causes. A further limitation is that the procedures in this study were performed by high-usage surgeons, which limits its generalisability. However, evidence suggests that if surgeons adhere to the recommended indications and surgical techniques, they get similar results [8]. Therefore, the conclusions of this study should relate to all surgeons using the recommended indications and techniques.

## Conclusion

This study demonstrates that at 5 years, both cemented and cementless UKR have remarkably low pain levels compared to TKR scores reported in literature. The cementless implant had significantly less pain than the cemented, with most patients experiencing no pain at all and no patients experiencing more than moderate pain. The physiological origin of chronic pain experienced after UKR remains unclear but at least some of the pain may not originate from the knee.
